# Identifying core symptoms of anxiety and depression in colorectal cancer patients: network analysis

**DOI:** 10.3389/fpsyt.2026.1892567

**Published:** 2026-08-24

**Authors:** Yu Chen, Jing Chen, Dandan Wu

**Affiliations:** 1 Department of Nursing, Jinzhou Medical University, Jinzhou, Liaoning, China; 2 Department of Nursing, Kunshan Hospital of Traditional Chinese Medicine, Kunshan, Jiangsu, China; 3 The First Affiliated Hospital of Jinzhou Medical University, Jinzhou, Liaoning, China

**Keywords:** anxiety, colorectal cancer, depression, latent profile, network analysis

## Abstract

**Background:**

Negative emotions, particularly anxiety and depression, are highly prevalent among cancer patients. Due to the unique disease characteristics of colorectal cancer, the coexistence of depression and anxiety is even more common, warranting close attention from clinical practitioners. This study aims to elucidate the characteristics of anxiety and depression symptom networks in patients with colorectal cancer.

**Methods:**

A cross-sectional study was conducted among 364 colorectal cancer patients recruited via convenience sampling in a hospital setting. Latent profile analysis was used to classify patients based on their anxiety and depression symptoms. Then network analysis was used to obtain the key nodes and compare the differences in the network structure between patients with and without stoma.

**Results:**

The mean scores for anxiety and depression were 5.93 and 7.23, respectively. Patients were classified into three subgroups. Regression analysis indicated that stoma status, diarrhea/constipation status, pain, and perceived stress significantly predicted subgroup membership. D2(depressed or sad mood) was identified as a key symptom in patients who require intervention, while D8(psychomotor agitation or retardation) served as a bridge symptom between anxiety and depression. Significant differences in network structure were observed between patients with and without an stoma.

**Conclusion:**

Both anxiety and depression were generally at mild levels, with depression severity slightly higher than anxiety. Targeting the identified central and bridge symptoms may help guide intervention efforts for colorectal cancer patients at risk of or suffering from anxiety and depressive symptoms, thereby contributing to the alleviation of these symptoms. Clinicians can use these findings to develop individualized treatment plans tailored to patient-specific needs.

## 1 Introduction

Colorectal cancer is currently the second leading cause of cancer-related deaths worldwide (1). Its incidence and mortality rates vary significantly across different populations, including gender, age, and race (2). It is estimated that by 2040, the number of new colorectal cancer cases globally will reach 3.2 million (3). Studies have shown that colorectal cancer primarily occurs in individuals over 50 years of age, with a higher prevalence in males than females (4). Approximately 1.9 million new cancer cases are diagnosed annually worldwide, with the traditional highest incidence rates observed in Western developed countries. However, the incidence of colorectal cancer is rapidly increasing among younger generations in both developing and developed countries and rises markedly with age (5). Unfortunately, colorectal cancer is often not detected at an early stage, resulting in most patients being diagnosed when the disease is advanced (6). During cancer treatment, patients may experience various adverse consequences, including economic toxicity (7), increased physical functional limitations (8), and treatment-related side effects (9), all of which can trigger significant negative emotions. Howren et al. (10) reported that colorectal cancer patients have a significantly higher likelihood of developing mental health disorders compared to the general population. Among these disorders, anxiety (prevalence range: 1.0% to 47.2%) and depression (prevalence range: 1.6% to 57.0%) are relatively common in colorectal cancer patients (11). Negative emotions such as anxiety and depression can cause severe psychological stress, reduce treatment adherence, affect prognosis, and further limit patients’ daily activities and quality of life (12, 13). Nevertheless, healthcare providers in China often prioritize cancer treatment while neglecting comorbid mental health conditions. Likewise, patients and their families tend to overlook mental health during the treatment process (14). Previous studies have proposed that anxiety and depression are highly comorbid and interrelated (15, 16), with comorbidity rates ranging from 10% to 90%. Patients presenting with both anxiety and depression often experience more severe conditions than those with only one disorder (17). Understanding the mechanisms underlying depression and anxiety in colorectal cancer patients is therefore crucial. By identifying relevant influencing factors and the mechanisms of symptom occurrence across different patient subgroups, more effective clinical interventions can be developed. Consequently, a systematic analysis of patients’ anxiety and depression status is necessary to inform appropriate intervention strategies.

Latent profile analysis (LPA) is a method that uses probabilistic estimation and comparison to identify distinct groups and their proportions, enabling the detection of population heterogeneity and the identification of subtypes based on different patterns of social support (18). However, the score of a single symptom cannot capture the interactions among symptoms. Some studies have noted that changes in one symptom can directly affect others (19). Network analysis addresses this limitation by identifying and visualizing complex relationships among multiple variables and pinpointing potential associated factors (20). It can effectively complement the psychological characterization of individuals within different latent profile subgroups, helping to elucidate the mechanisms of key symptoms that exert strong influences on other nodes. This, in turn, facilitates the development of more effective interventions or novel support models aimed at enhancing patients’ sense of security and confidence in recovery (21). In this study, LPA was first employed to reduce the patient dimension and reveal heterogeneous subpopulations within the sample. Subsequently, network analysis was performed on the identified subgroups to explore the complex interaction patterns among factors within each subgroup from a relational perspective. By comparing the network structures across different profiles, a multidimensional analysis can be relied upon to understand the mechanisms. Personalized and accurate identification of patients’ anxiety and depression status can provide more precise intervention targets for symptom management and scientific intervention processes in high-risk groups, thereby improving rehabilitation outcomes and patients’ psychological resilience.

Previous studies have divided subjects into three distinct subgroups characterized by low, moderate, and high levels of depression and anxiety (22). According to Liu et al. (23),based on different profile groupings, the core symptoms in the low and moderate symptom groups were “sadness or pain” and “anxiety or worry,” whereas the core symptoms in the high symptom group were “crying” and “uncontrollable worry.” Due to factors such as sex hormone secretion, early life experiences, and social role expectations, women are generally considered more prone to depression and anxiety than men (8, 24). However, most of these studies have focused on other specific populations, leaving a research gap regarding anxiety and depression specifically in colorectal cancer patients. Given that the patterns and clinical features of depression and anxiety may vary depending on sample characteristics—such as age, culture, socioeconomic status, presence or absence of medical conditions, and unique stressors—it is not valid to assume that network analysis results from one population apply to others. Therefore, the network structure of anxiety and depressive symptoms should be analyzed separately within specific populations (19). Accordingly, this study opted to identify subgroups requiring intervention and their key symptom nodes by combining latent profile analysis with network analysis, thereby assisting healthcare professionals in implementing symptom severity-stratified interventions and developing personalized management strategies. The objectives of this study were to: (1) examine the overall levels of anxiety and depression in colorectal cancer patients; (2) accurately identify latent profiles and analyze their influencing factors; (3) identify the core symptoms of high-risk groups through network analysis and detect bridge nodes connecting anxiety and depression clusters; and (4) explore whether differences exist in global strength and network structure between patients with and without a stoma by comparing their network diagrams.

## 2 Methods

### 2.1 Design and participants

As a cross-sectional study, a total of 364 patients with colorectal cancer were recruited from tertiary hospitals in Liaoning Province between March 2026 and May 2026 using a convenience sampling method. Patients were included if they met the following criteria: (1) pathologically confirmed diagnosis of colorectal cancer; (2) provided informed consent and voluntarily participated in the study; (3) possessed adequate comprehension and no communication barriers, enabling them to complete the questionnaires. Patients were excluded if they met any of the following criteria: (1) presence of severe acute or chronic diseases (e.g., cardiac, pulmonary, or renal failure); (2) exposure to stressful life events (e.g., family upheavalst, emotional trauma) within the past month; (3) mental disorders that prevented cooperation with the survey; (4) refusal to participate in the study.

All participants signed informed consent forms prior to enrollment. This study was approved by the Ethics Committee of Jinzhou Medical University (JZMULL2026097) and was conducted in accordance with the Declaration of Helsinki and its subsequent amendments. All participants were ensured to have signed written informed consent and were informed of their right to withdraw from the study at any time. The reporting of study results followed the Strengthening the Reporting of Observational Studies in Epidemiology (STROBE) statement. Data were collected through a combination of paper-based questionnaires and an online survey platform (Questionnaire Star application) distributed to colorectal cancer patients who met the inclusion criteria. Members of the research team received topic-specific training and became familiar with each patient’s medical history, data collection procedures, and related precautions. Prior to questionnaire administration, the researchers explained the purpose of the study, the required time commitment, confidentiality measures, questionnaire instructions, and the participants’ right to withdraw at any time, using concise and easy-to-understand language. The researchers also guaranteed that all patient-identifiable information would be kept confidential during subsequent data analysis and manuscript writing, and that the questionnaire results would be used solely for academic purposes, without any commercial exploitation or privacy breaches. All patient inquiries were thoroughly addressed before participants voluntarily decided whether to sign the informed consent form. Participants were then asked to complete the study questionnaires in a quiet room. The questionnaires were self-administered by the patients and took approximately 15 minutes to complete.

During the data collection process, if patients had difficulty understanding any item, the researchers provided assistance and explanations using standardized, non-leading language. While ensuring scientific accuracy, the researchers paid close attention to the neutrality and sensitivity of wording, avoiding any stimulating or suggestive terms that might cause psychological discomfort or emotional distress, and particularly refrained from using sensitive expressions to minimize any potential impact on the patients’ psychological state. Disease-related information was completed by the investigators based on electronic medical records, while the remaining assessment tools were completed by the patients. This study fully respected the participants’ personal wishes, and the researchers continuously monitored patients’ responses during data collection. If a patient exhibited signs of distress, restlessness, or other emotional disturbances, the study procedures were immediately paused or completely terminated, and psychological support resources were provided. A small gift was given to each patient upon completion of the questionnaires as a token of appreciation. Upon completion, the questionnaires were immediately reviewed, and participants were asked to fill in any missing items before on-site collection. A double-check procedure was employed to ensure data accuracy. The researchers stored the data appropriately and subsequently destroyed it after the retention period expired to protect patient privacy.

### 2.2 Sample size

For LPA, Nylund-Gibson and Choi (25)suggested that the minimum sufficient sample size standard for LPA should be 300 cases. During the data collection phase, 376 questionnaires were distributed. A total of 12 questionnaires were excluded after the survey, and 364 questionnaires were successfully collected. We used G*POWER software for sample size estimation. The parameters were set as follows: effect size =0.15, significance level α=0.05, power =95%, and finally 134 cases were calculated. An additional 20% invalid response rate was included, resulting in 173 samples needed. The final sample size for this study met the strict criteria established for the analysis.

### 2.3 Measures

The patients’ general conditions (including age, gender, smoking and drinking, insomnia, etc.) and medical conditions (including time of diagnosis, tumor stage, stoma, radiotherapy and chemotherapy, etc.) were included.

#### 2.3.1 Generalized Anxiety Disorder-7

The GAD scale was developed by Spitzer (26) and cited 34 to screen cases of generalized anxiety disorder and assess the severity of anxiety symptoms. It contains a total of 7 items. GAD-7 was scored on a four-point Likert-type scale ranging from 0 (not at all) to 3 (almost every day). The GAD-7 scores range from 0 to 21, with higher scores indicating greater anxiety. A GAD-7 score of 0–4 indicates no anxiety, and a GAD-7 score of 5 or more indicates anxiety (5–9 points as “mild anxiety,” 10–14 points as “moderate anxiety,” and 15–21 points as “severe anxiety”). The GAD-7 scale has been proved to be reliable in Chinese inpatients, and in the current study of colorectal cancer patients, the Cronbach’s α coefficient was 0.705.

#### 2.3.2 Patient Health Questionnaire-9

The PHQ-9, developed by Kroenke (27), is a reliable and valid 9-item measure of self-reported depression severity. Each item of the PHQ-9 is rated on a four-point Likert scale ranging from 0 (not at all) to 3 (almost every day). The total score on the PHQ-9 ranges from 0 to 27, with higher scores indicating more severe depression. 37PHQ-9 scores of 0–4 indicated no depression and ≥5 indicated depression (5-9: mild depression; 10-14: moderate depression; 15-27: moderate-to-severe depression). In the present study, the Cronbach’s α coefficient was 0.711.

#### 2.3.3 Pain Catastrophizing Inventory (PCS)

PCS assessment tends to produce excessive negative thinking during anticipatory or actual pain (Sullivan et al., 1995) (28). The scale contained 13 items, and participants rated each item on a 5-point Likert scale ranging from 0= “not at all” to 4= “all the time”. Total scores range from 0 to 52 (rumination range, 0 to 16; amplification range, 0 to 12; helplessness range, 0 to 24), with higher scores indicating greater catastrophizing of pain. In the present study, the Cronbach’s α coefficient was 0.903.

#### 2.3.4 Perceived Stress Scale

This scale was compiled by American researchers Cohen et al. (29) in 1983. It is widely used in the world to measure the perceived stress or stress level of individuals. The scale consisted of 10 items, each item was scored from 1 to 4, and the total score ranged from 4 to 16. Higher scores indicate higher levels of perceived stress by individuals. In the present study, the Cronbach’s α coefficient was 0.749.

### 2.4 Data analysis

All questionnaire data were entered and verified by two independent researchers and then categorized using Excel software. Statistical analyses were performed using SPSS 26.0, Mplus 8.3, and R language. Anxiety and depression scores were included as explicit variables. LPA was used to explore the optimal number of profiles. The analysis began with a single-class model and progressively increased the number of classes until the model fit indices reached optimal levels. Model fit was evaluated using the following indices: Akaike Information Criterion (AIC), Bayesian Information Criterion (BIC), adjusted BIC (aBIC), likelihood ratio test (LRT), bootstrap likelihood ratio test (BLRT), and entropy. Lower values of AIC, BIC, and aBIC indicated better model fit. LRT and BLRT were used to assess the absolute fit between the K-1-class model and the K-class model. Entropy was evaluated as an indicator of classification accuracy, with an entropy value greater than 0.80 indicating sufficient classification accuracy. SPSS 26.0 software was used for data analysis. Normality of all continuous variables, including sociodemographic data and continuous scale scores, was tested using the Shapiro–Wilk test. Data were presented as frequencies and percentages, and comparisons were made using the chi-square (χ²) test. In the univariate analysis, the Bonferroni correction was applied to control for Type I error inflation across the 19 variables tested for between-group differences, with the adjusted significance threshold set at p < 0.0026 (0.05/19). Only variables meeting this criterion were entered into the subsequent multinomial logistic regression analysis. Model fit was assessed using the model fitting information test, and explanatory power was reported via Cox & Snell’s R² and Nagelkerke’s R². Effect sizes for each predictor are presented as regression coefficients (B), standard errors (S.E.), Wald χ² values, odds ratios (OR), and their 95% confidence intervals (CIs). Statistical significance was set at p < 0.05.

The anxiety-depression subgroups were then included as a whole in the network analysis. The qgraph package in R was used to establish the anxiety and depression network model for patients (30). All symptom networks were estimated using the partial correlation network method. A sparse Gaussian graphical model based on partial correlations was constructed using the least absolute shrinkage and selection operator (lasso) regularization method, which shrinks all edges and small edge sets in the network precisely to zero. The gamma hyperparameter was set to 0.25 (31). In the network, each node (circle) represents a single item. An edge, depicted as a line connecting two nodes, represents the partial correlation coefficient, indicating the regularized relationship. Green lines indicate positive correlations, while red lines indicate negative correlations. Edge weights are represented by the thickness of the lines; thus, stronger correlations correspond to thicker lines. The network was visualized using the Fruchterman–Reingold algorithm (28), in which nodes that are more strongly and frequently associated with each other are positioned closer together and appear more concentrated in the network. In this study, core symptoms were identified using the strength centrality index, as previous research has demonstrated that strength is more stable and reproducible than closeness and betweenness centrality, which tend to exhibit greater instability and limited interpretability and practicality in psychological networks (32, 33). Core symptoms can serve as useful and effective potential intervention direction for developing therapeutic interventions. The network tools package in R was used to evaluate bridge nodes in the anxiety and depression network of colorectal cancer patients. Bridge expected influence (BEI) centrality was selected, as BEI is considered a more appropriate centrality measure for identifying bridge nodes in networks containing both positive and negative relationships (34). BEI is defined as the sum of edges connecting a given node to all nodes in the opposing community. A higher BEI value indicates a stronger connection with nodes in the other community (35). Given the potential impact of stoma status on anxiety and depressive symptoms in colorectal cancer patients (36, 37), the anxiety-depression networks of the stoma and non-stoma groups were compared. Network Comparison Test (NCT) was conducted using the Network Comparison Test package in R (38). Networks were estimated using the EBICglasso method (gamma = 0.25) with 1,000 permutations. The following indices were examined (1): global strength invariance; and (2) network structure invariance (maximum edge weight difference, M statistic). Statistical significance was set at p < 0.05.

The bootnet package was used to assess the stability of the network model. Network stability was evaluated using a bootstrap procedure with 1,000 iterations to compute the accuracy and stability estimates of network edges. The edge weights was estimated with 95% confidence intervals (CIs) obtained via bootstrapping. Overlap between these confidence intervals indicates low accuracy. The stability of node centrality indices was examined using a case-dropping subset bootstrap procedure. The centrality stability (CS) coefficient was used as a reference index. A CS coefficient equal to 0.25 indicates acceptable stability, while a value greater than 0.5 indicates relatively good stability (39).

## 3 Results

### 3.1 Sample size characteristics

All scale data were collected on-site and checked by the study staff, ensuring a 100% completion rate and no missing data. A total of 376 questionnaires were distributed after informed consent was obtained from patients between March 2025 and May 2026. Due to the presence of contradictory information in the responses, 12 questionnaires were excluded, 364 cases were finally included, and the effective recovery rate was 97.33%. The average scores of anxiety and depression were 5.93 and 7.23, respectively, which were at the level of mild symptoms.

### 3.2 Classification of potential profiles

**Figure 1:**
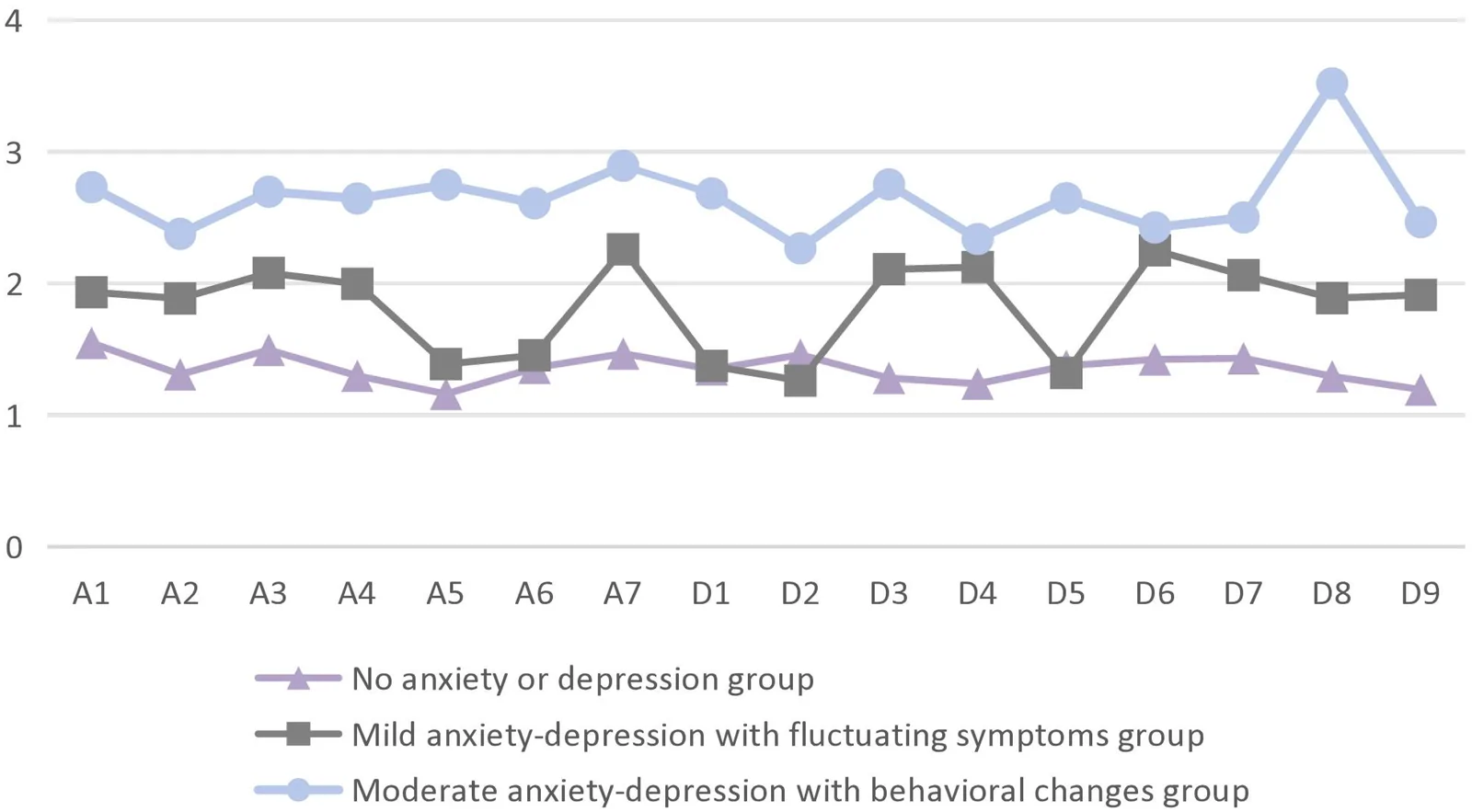
The potential distribution of anxiety and depression in patients with colorectal cancer.

The information-based fitting index shows the classification situation from category 1 to category 5 (**Table 1**). It can be seen that AIC, BIC, and aBIC are gradually decreasing. The decreasing trend slows down in the fourth category and the entropy was lower than that of the 3-class solution, resulting in exclusion. Based on a comprehensive consideration of model simplicity, statistical robustness, and interpretability, the three-category model is chosen as the better one. The three subgroups are shown in **Figure 1**. They account for 27.747% (n = 101), 56.868% (n = 207), and 15.385% (n = 56) respectively. The average score of the items in the first group is the lowest among the three groups and the scores of each item are higher than the average. According to the scoring standard of the scale, patients in this subgroup do not have anxiety or depression, so it is named the no anxiety or depression group. The overall score of the second group was at a moderate level, but the scores of individual symptoms (A5,A6,D1,D2,D5) showed significant fluctuations. Therefore, it is named the mild anxiety-depression with fluctuating symptoms group. The last group has the highest level of anxiety and depression, and the score of D8 “psychomotor agitation or retardation” is significantly higher than other items, so it is named the moderate anxiety-depression with behavioral changes group.

**Table 1:** Potential overview analysis of the model fitting index.

Profile	AIC	BIC	aBIC	BLRT(p)	VLMR(p)	Entropy	Proportion min
1	13285.590	13410.299	13308.776	\	\	\	\
2	11947.279	12138.239	11982.783	0.000	0.000	0.998	15.385
3	11511.952	11769.164	11559.774	0.000	0.000	0.952	15.385
4	11393.700	11717.163	11453.840	0.518	0.522	0.926	13.187
5	11312.350	11702.065	11384.807	0.764	0.765	0.930	6.319

### 3.3 Different potential types of influencing factors

This study employed the χ^2^ test to compare the differences among different subgroups. The results of the statistical test indicated that there are differences among the various categories in terms of age, chronic disease status, etc. (p < 0.05). In contrast, other factors such as gender, marital status, and average income did not show significant statistical differences (p > 0.05) (**Supplementary Table 1**).

In **Table 2**, a logistic regression analysis was further conducted to explore the predictive factors. The three potential subgroups were used as dependent variables. Variables that were statistically significant in the univariate analysis were included as independent variables. Compared with the moderate anxiety-depression with behavioral changes group, patients with stoma and constipation/diarrhea were less likely to be assigned to the no anxiety and depression group; patients with higher pain catastrophizing and perceived stress scores were more likely to be assigned to the moderate anxiety-depression with behavioral changes group. Compared with the moderate anxiety-depression with behavioral changes group, patients with stoma, higher pain catastrophizing and perceived stress scores were less likely to be assigned to the mild anxiety-depression with fluctuating symptoms group. The model yielded a Cox & Snell R² of 0.679 and a Nagelkerke R² of 0.794, indicating that the model explained approximately 79.4% of the variance in the outcome variable, suggesting strong explanatory power.

**Table 2:** Multiple regression analysis of latent features.

Moderate anxiety-depression with behavioral changes group(ref)vs No anxiety or depression group						
	β	SE	Wald χ^2^	P	OR	95%CI
Does the patient have an stoma?						
Yes	-5.059	0.957	27.947	<0.001	0.006	0.001-0.041
Is there any case of constipation/diarrhea?						
Yes	-2.283	1.129	4.091	0.043	0.102	0.011-0.932
**Pain**	-0.601	0.074	65.277	<0.001	0.548	0.474-0.634
**Perception of stress**	-0.512	0.15	11.595	0.001	0.599	0.446-0.805

Moderate anxiety-depression with behavioral changes group(ref)vs Mild anxiety-depression with fluctuating symptoms group						
	β	SE	Wald χ^2^	P	OR	95%CI
Does the patient have an stoma?						
Yes	-4.022	0.863	21.719	<0.001	0.018	0.003-0.097
**Pain**	-0.289	0.063	21.153	<0.001	0.749	0.662-0.847
**Perception of stress**	-0.433	0.123	12.318	<0.001	0.648	0.509-0.826

### 3.4 Network analysis graph and centrality index

**Figure 2:**
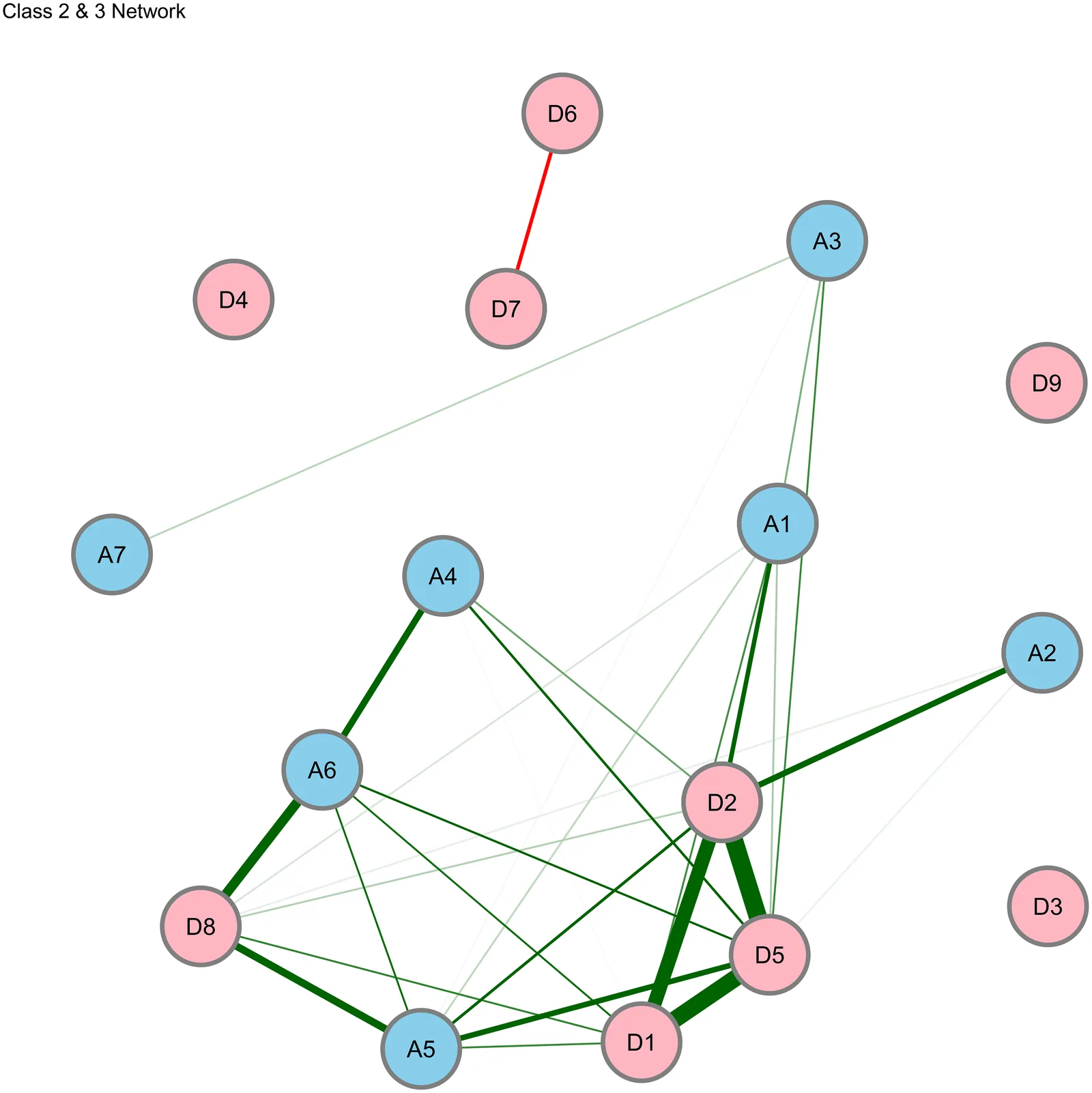
Network analysis diagram of patients with anxiety and depression among patients with colorectal cancer.

**Figure 3:**
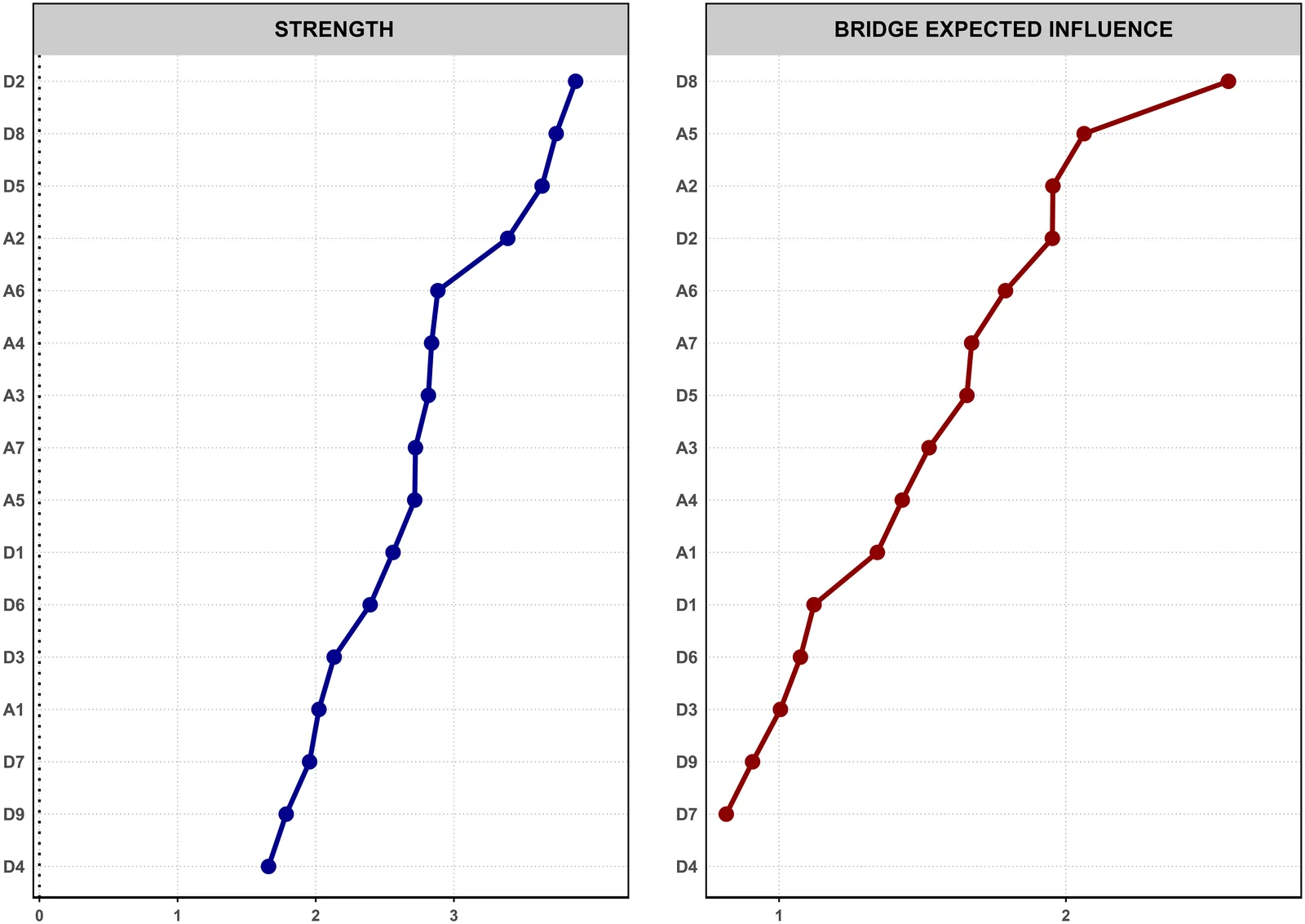
Strength indicators and bridge expected influence.

**Figure 2** depicts the symptom network of patients with anxiety and depression (mild anxiety-depression with fluctuating symptoms group and moderate anxiety-depression with behavioral changes group), with green lines showing positive correlations and red lines showing negative correlations. In the network diagram, the combinations with the highest correlation coefficients were D2 - D5(r=0.237),D1 - D5(r=0.222) and D1 - D2(r=0.191). **Figure 3** represents the intensity centrality index, and the nodes with the highest intensity can be regarded as the core symptom of this network, as they have the most significant effect on every node in the network. Among them, D2 (r_s_= 3.881) had the highest intensity index, followed by D8(r_s_=3.740) and D5 (r_s_= 3.638). These nodes are most highly correlated with other nodes in the subgroup network and can be important potential intervention direction for specifying interventions within different populations. D8 (r_s_=2.567), A5 (r_s_=2.063), and A2 (r_s_=1.955) were the symptoms with the highest BEI in these two communities and were identified as bridge symptoms.

**Figure 4:**
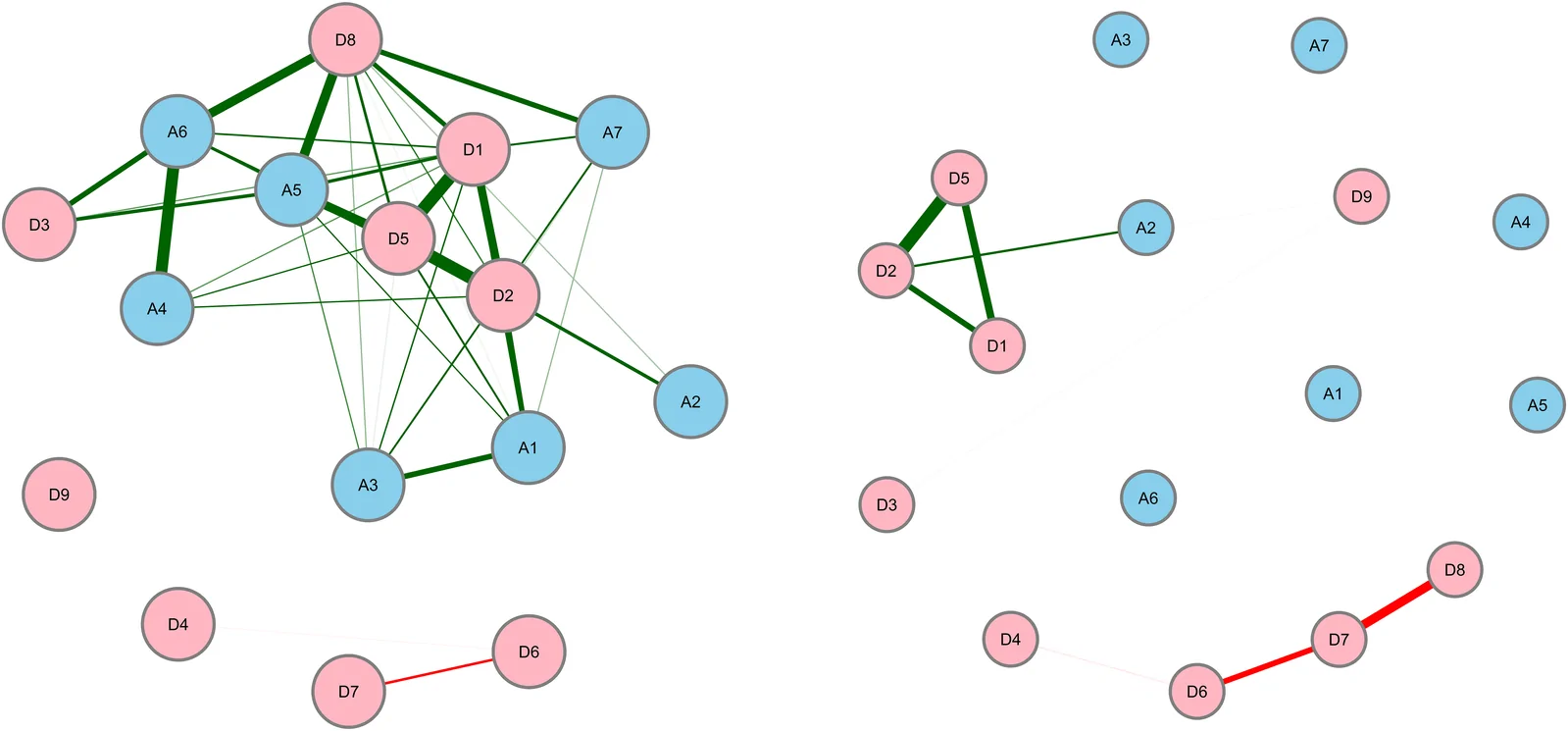
Network diagram of patients with and without stoma devices. (Left picture: stoma exists; Right picture: No stoma).

**Figure 5:**
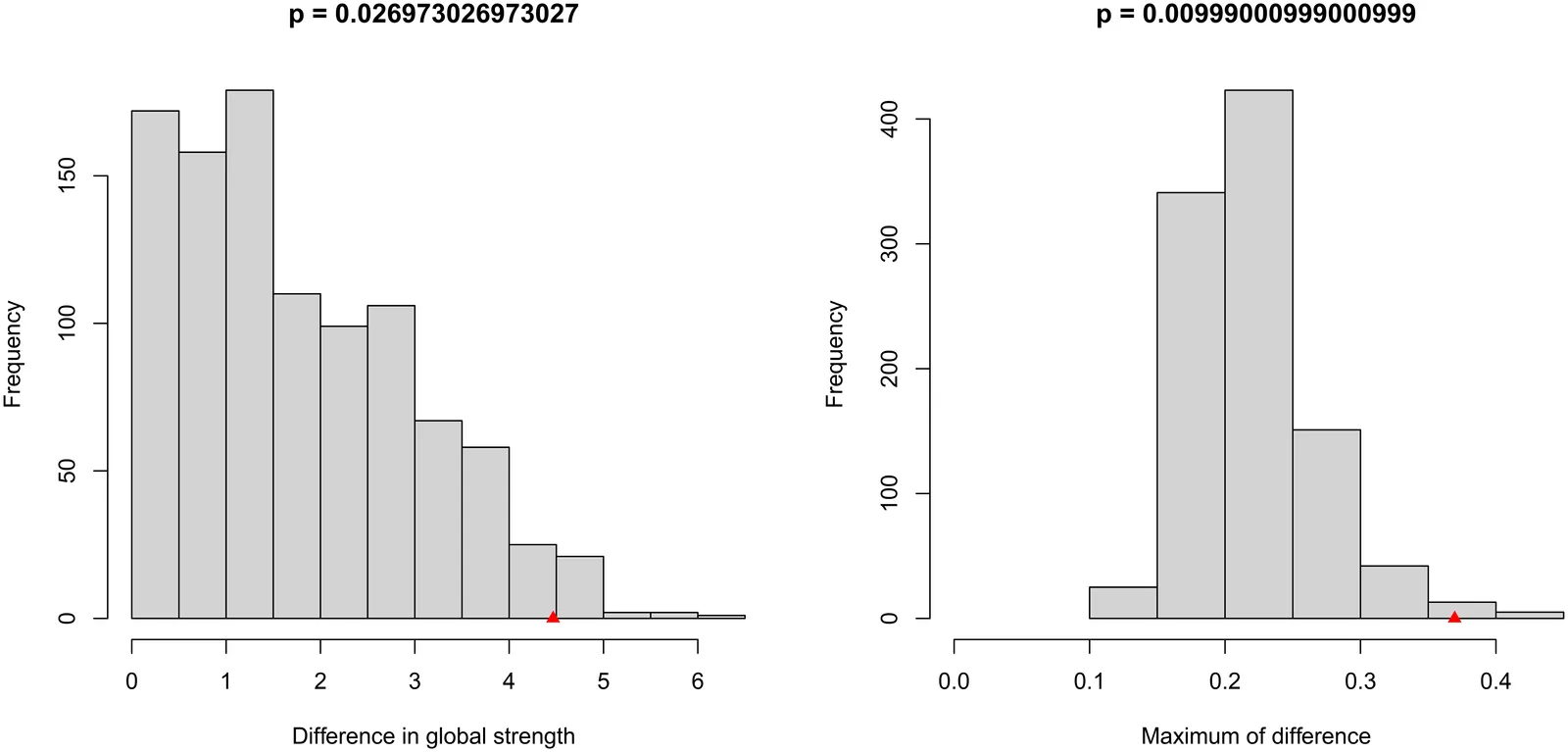
Distribution of network differences between patients with and without stoma based on arrangement.

In the network analysis according to stoma in **Figure 4**, the most relevant links in the network structure of patients with stoma were D2 - D5(r=0.229), D1 - D5(r=0.228) and D4 - D6(r=0.216). Without a colstoma for D2 - D5 (r = 0.151), D7 - D8 (r = 0.119) and D1 - D5 (r = 0.106). The node with the highest intensity in patients with stoma was D5(r_s_=0.946). D2(r_s_=0.249) was the key symptom of absence of stoma (**Supplementary Figure 1**). The Network Comparison Test (NCT) revealed a significant difference in global strength between the stoma and non-stoma groups (difference = 4.47, 95% permutation interval: 0.09~4.50, p = 0.027), with the stoma group exhibiting significantly higher overall connectivity. The network structures also differed significantly between the two groups (maximum edge weight difference M = 0.37, the 95% range of the permutation null distribution: 0.15~0.34, p = 0.010), indicating distinct symptom network structure between patients with and without a stoma (**Figure 5**).

The stability of the study showed satisfactory results. As shown in **Supplementary Figures 2**–**5**, the width of the 95% confidence interval of the edge weights of the symptom networks in each group was narrow, indicating that the estimated value of the edge weights had high accuracy, which further verified that the estimation accuracy of the network connection strength was reliable. The associated stability coefficients (CS coefficients) of key network strength measures for subgroup and stoma condition showed significant performance (CS≥0.7), and the bridge expected influence CS index >0.5.

## 4 Discussion

Clinically, psychological research has often been variable-centered. This approach assumes that individuals are drawn from a homogeneous population and estimates parameters as “averaged” effects across the sample. However, this assumption oversimplifies empirical data and underestimates the possibility that individuals may exhibit unique patterns of behavior, attitudes, or experiences, thereby forming distinct latent subgroups (40). The present study adopted a person-centered approach using LPA to distinguish individuals with similar characteristics into distinct classes with meaningful features and to analyze the characteristics of each latent class. Subsequently, anxiety and depression networks were constructed to highlight key symptoms within patients, providing theoretical evidence for the development of personalized and precise symptom management strategies. Among the three derived subgroups, the mild anxiety-depression with fluctuating symptoms group and the moderate anxiety-depression with behavioral changes group accounted for 56.868% and 15.385% of the sample, respectively. The no anxiety-depression group showed the most favorable outcome, representing 27.747% of the total sample.

In this study, anxiety and depression were found to be at mild levels, which is consistent with the findings of previous research (41). The presence of a stoma, even if temporary, exacerbates anxiety and depression in patients through multiple pathways. On a physical level, common complications such as skin erosion, stoma necrosis, prolapse, parastomal hernia, and dehydration directly impair physical health and quality of life, while continuously reinforcing patients’ sense of loss of control over their illness (37). Psychologically, insufficient preoperative education, inadequate stoma care skills, and maladjustment readily trigger anger, fear, severe anxiety, and depression. Even after stoma reversal or completion of chemotherapy, colorectal cancer patients exhibit significantly higher levels of anxiety and depression than healthy populations (36). On a social level, patients frequently experience sexual dysfunction, inconvenience in dressing and going out, and concerns about stomal noise and visible appearance. These limitations further reduce social participation and weaken social support systems. In summary, the presence of a stoma not only brings about physical changes but also creates a vicious cycle through the multidimensional factors described above, substantially intensifying psychological symptoms. Therefore, clinical interventions should address the management of physiological complications, psychological adaptation support, and social function rehabilitation to alleviate the long-term psychological burden on patients. Consistent with the findings of Johnson et al. (42), constipation and diarrhea are correlated with anxiety and depression. The presence of constipation and diarrhea persistently disrupts physical comfort and fecal controllability, inducing anticipatory anxiety about incontinence and social embarrassment. The unpredictability of symptoms impairs daily routine, reduces self-efficacy, and consequently contributes to depressive mood. This physiological-psychological negative cycle represents an important mechanism underlying the high prevalence of anxiety and depression. Previous research (43) has further suggested that, in the same disease context, anxiety is associated only with diarrhea, whereas depression is a typical manifestation of constipation. Our study did not explore these specific relationships, which could be investigated in future research.

Pain, along with the use of analgesic medications (44) and other factors, may render patients more susceptible to negative expectations about the future, thereby exacerbating anxiety and depression. Persistent stress, in turn, depletes an individual’s psychological coping resources, leading to a sense of powerlessness to change the current situation and consequently enhancing feelings of helplessness and low mood. When pain and stress co-occur, pain continuously triggers emotional distress, while stress weakens the capacity to regulate these emotions, creating a vicious cycle that makes anxiety and depression difficult to alleviate.

To the best of our knowledge, this study is the first to investigate the anxiety-depression symptom network in patients with colorectal cancer. In this study, D2 (depressed or sad mood) exhibited the highest network strength value and was the most central symptom in the entire network, followed by D8 (psychomotor agitation or retardation). Early detection and alleviation of negative emotional distress are key components of effective symptom management.

Strength centrality reflects the direct connections of a node within the network, measured as the sum of the absolute edge weights connecting it to all other nodes. Higher strength indicates greater importance within the network (45). As the core symptom of the subgroup of patients with anxiety and depression (17), D2 (depressed or sad mood) suggests that the core problem in these patients is characterized by persistent low mood, pessimistic expectations regarding their own condition, and difficulty experiencing pleasure or hope in daily activities. These manifestations not only directly reflect patients’ psychological distress but are also closely linked to anxiety symptoms, with the two mutually reinforcing and amplifying each other. Colorectal cancer patients must confront threats to their personal survival while accepting the reality of impaired self-image. This is particularly evident among younger patients, who are especially prone to negative emotions (46). Additionally, chemotherapy imposes a substantial financial burden; patients often worry that the high treatment costs will increase their family’s economic strain and that their illness may disrupt family harmony (47). The most prominent bridge symptom connecting anxiety and depression in this sample was D8 (psychomotor agitation or retardation), which aligns with the findings of Kaiser et al. (48). Bridge symptoms increase the risk of interactions between different disorders. D8 reflects observable behavioral changes, including marked slowing of movements or speech, or, conversely, restlessness and excessive body movements. Helgadóttir et al. (49) suggested that patients with comorbid depressive and anxiety disorders were more active than those with either condition alone. Identifying bridge symptoms may help clarify the complexity of comorbidity and facilitate exploration of the mechanisms underlying the co-occurrence of anxiety and depression symptom communities (50).

Following group-specific network comparisons based on stoma status, significant differences in the core symptom structure were observed between the two groups. For colorectal cancer patients without a stoma, the core symptom remained D2 (depressed or sad mood), which is consistent with the general psychological response pattern in colorectal cancer patients, wherein negative emotional experiences driven by the disease itself dominate the symptom network. However, for patients with a stoma, the core symptom shifted to D5 (poor appetite or overeating). A possible explanation for this shift is that the presence of a stoma directly alters patients’ eating and defecation rhythms. Patients may deliberately adjust the type and amount of food they consume due to concerns about stomal gas, leakage, or uncontrollable defecation (51), leading to appetite disturbances. Concurrently, stoma-related changes in body image and feelings of social embarrassment may also influence eating behaviors (52). Adam et al. (53) suggested that acute stress may transiently suppress appetite through activation of the sympathetic-adrenomedullary system, whereas chronic stress may promote increased eating behavior via sustained activation of the HPA axis. A bidirectional association may exist between dietary changes and emotional distress: emotional distress may be accompanied by alterations in eating behavior, while disordered eating may in turn exacerbate emotional burden, forming a vicious cycle of “stress–eating–emotion” (54, 55). To date, no study has directly examined the central role of ‘poor appetite or overeating’ within the anxiety-depression network of colorectal cancer patients with a stoma, and this issue warrants further investigation in future research. In the stoma group, eating-related distress becomes the most prominent, most controllable, and most easily disrupted aspect of daily life, thus conferring the highest strength to this symptom within the network.

Therefore, interventions targeting key nodes such as anxiety, sadness, behavioral changes, and appetite alterations may contribute to the prevention and alleviation of anxiety and depressive symptoms in colorectal cancer patients. Clinicians should attend not only to patients’ emotional and behavioral changes but also to the assessment and regulation of eating behaviors, thereby reducing patients’ anxiety and depression levels in a targeted and systematic manner.

First, given the cross-sectional design used to construct the network model, the centrality indices reflect the strength of associations among nodes within the network rather than direct causal relationships (56, 57). Future research should integrate longitudinal designs to validate the findings. Second, the sample was drawn from a single province via convenience sampling, which may limit generalizability to other regions or healthcare systems. Thirdly, the sample size of this study is limited, which may affect the stability of the network structure. Therefore, the results should be interpreted with caution. Finally, the relatively low Cronbach’s α coefficients for the GAD-7 (α = 0.705) and PHQ-9 (α = 0.711) may introduce measurement error, potentially affecting edge weight estimation and the stability of the network structure. Future research should incorporate multi-center samples and measurement instruments with higher reliability to validate these findings.

## 5 Conclusion

To avoid information loss caused by artificially set thresholds while significantly improving the precision of group classification, this study employed LPA to identify subgroups among patients with colorectal cancer. Network analysis was then conducted on patients presenting with anxiety and depression symptoms to identify key symptomatic nodes, thereby providing a theoretical reference for healthcare professionals in developing intervention strategies. We recommend targeting depressed or sad mood to alleviate negative emotions and psychological distress in colorectal cancer patients. A comprehensive clinical management approach should be established to help patients achieve more optimal levels of resilience and improved rehabilitation outcomes.

## Data Availability

The raw data supporting the conclusions of this article will be made available by the authors, without undue reservation.
