# Supplementary material for: Identifying core symptoms of anxiety and depression in colorectal cancer patients: network analysis
Source: Front Psychiatry. 2026 Aug 24;17:1892567. doi: 10.3389/fpsyt.2026.1892567 (PMC13547281; doi:10.3389/fpsyt.2026.1892567)
Supplement: Supplementary file 1 [file Supplementaryfile1.docx]

Supplementary Material

**Table.S1** Results of univariate analysis of patients

|  | No anxiety or depression group | Mild anxiety and depression-unstable group | Moderate anxiety depression-behavior change group | χ2 | P |
| --- | --- | --- | --- | --- | --- |
| Age |  |  |  | 42.319^a^ | <0.001 |
| < 30 | 25(24.80%) | 96(46.40%) | 44(78.60%) |  |  |
| 31 - 60 | 76(75.20%) | 111(53.60%) | 12(21.40%) |  |  |
| Gender |  |  |  | 3.375^a^ | 0.185 |
| Man | 60(59.40%) | 101(48.80%) | 27(48.20%) |  |  |
| Woman | 41(40.60%) | 106(51.20%) | 29(51.80%) |  |  |
| Educational level |  |  |  | 5.896^b^ | 0.403 |
| Junior high school and below | 79(78.20%) | 141(68.10%) | 41(73.20%) |  |  |
| High school or technical school | 19(18.80%) | 51(24.60%) | 11(19.60%) |  |  |
| University or junior college | 3(3.00%) | 11(5.30%) | 2(3.60%) |  |  |
| University and above | 0(0.00%) | 4(1.90%) | 2(3.60%) |  |  |
| Occupational Status |  |  |  | 35.901^a^ | <0.001 |
| On the job | 19(18.80%) | 69(33.30%) | 37(66.10%) |  |  |
| Out of service | 82(81.20%) | 138(66.70%) | 19(33.90%) |  |  |
| Marital status |  |  |  | 3.776^b^ | 0.721 |
| Married | 78(77.20%) | 142(68.60%) | 42(75.00%) |  |  |
| Divorced | 10(9.90%) | 34(16.40%) | 6(10.70%) |  |  |
| unmarried | 1(1.00%) | 4(1.90%) | 1(1.80%) |  |  |
| Widowed | 12(11.90%) | 27(13.00%) | 7(12.50%) |  |  |
| Average household income |  |  |  | 3.702^b^ | 0.465 |
| <1500 | 60(59.40%) | 110(53.10%) | 32(57.10%) |  |  |
| 1500 - 3000 | 35(34.70%) | 90(43.50%) | 23(41.10%) |  |  |
| >3000 | 6(5.90%) | 7(3.40%) | 1(1.80%) |  |  |
| Is there a family history of gastrointestinal diseases? |  |  |  | 6.093^a^ | 0.050 |
| Yes | 63(62.40%) | 100(48.30%) | 33(58.90%) |  |  |
| No | 38(37.60%) | 107(51.70%) | 23(41.10%) |  |  |
| Tumor stage |  |  |  | 4.489^a^ | 0.355 |
| Stage I | 18(17.80%) | 36(17.40%) | 7(12.50%) |  |  |
| Stage II | 52(51.50%) | 90(43.50%) | 23(41.10%) |  |  |
| Stage III/IV | 31(30.70%) | 81(39.10%) | 26(46.40%) |  |  |
| Do you suffer from insomnia? |  |  |  | 4.079^a^ | 0.130 |
| Yes | 55(54.50%) | 115(55.60%) | 39(69.60%) |  |  |
| No | 46(45.50%) | 92(44.40%) | 17(30.40%) |  |  |
| Religious belief |  |  |  | 6.516^a^ | 0.037 |
| No | 86(85.10%) | 181(87.40%) | 55(98.20%) |  |  |
| Yes | 15(14.90%) | 26(12.60%) | 1(1.80%) |  |  |
| Do you live with your family? |  |  |  | 2.372^a^ | 0.302 |
| Yes | 76(75.20%) | 162(78.30%) | 48(85.70%) |  |  |
| No | 25(24.80%) | 45(21.70%) | 8(14.30%) |  |  |
| Type of medical insurance |  |  |  | 4.749^b^ | 0.583 |
| Employee Medical Insurance | 82(81.20%) | 160(77.30%) | 44(78.60%) |  |  |
| Resident Medical Insurance | 13(12.90%) | 27(13.00%) | 9(16.10%) |  |  |
| Self-payment | 2(2.00%) | 10(4.80%) | 3(5.40%) |  |  |
| Agricultural medical insurance | 4(4.00%) | 10(4.80%) | 0(0.00%) |  |  |
| Main caregiver |  |  |  | 6.198^a^ | 0.401 |
| Spouse | 79(78.20%) | 141(68.10%) | 41(73.20%) |  |  |
| Parents or children | 19(18.80%) | 51(24.60%) | 11(19.60%) |  |  |
| Other relatives or friends | 3(3.00%) | 11(5.30%) | 2(3.60%) |  |  |
| Other circumstances | 0(0.00%) | 4(1.90%) | 2(3.60%) |  |  |
| Diagnosis time |  |  |  | 2.695^a^ | 0.022 |
| < One month | 12(11.90%) | 23(11.10%) | 4(7.10%) |  |  |
| One month to three months | 39(38.60%) | 86(41.50%) | 19(33.90%) |  |  |
| More than three months | 50(49.50%) | 98(47.30%) | 33(58.90%) |  |  |
| Chronic disease condition |  |  |  | 7.382^a^ | 0.024 |
| Yes | 66(65.30%) | 136(65.70%) | 47(83.90%) |  |  |
| No | 35(34.70%) | 71(34.30%) | 9(16.10%) |  |  |
| Is there an stoma? |  |  |  | 100.571^a^ | <0.001 |
| Yes | 18(17.80%) | 61(29.50%) | 53(94.60%) |  |  |
| No | 83(82.20%) | 146(70.50%) | 3(5.40%) |  |  |
| Are you troubled by constipation or diarrhea? |  |  |  | 34.120^a^ | <0.001 |
| Yes | 51(50.50%) | 148(71.50%) | 53(94.60%) |  |  |
| No | 50(49.50%) | 59(28.50%) | 3(5.40%) |  |  |
| Chemotherapy and radiotherapy situation |  |  |  | 1.135^b^ | 0.645 |
| Yes | 3(3.00%) | 6(2.90%) | 3(5.40%) |  |  |
| No | 98(97.00%) | 201(97.10%) | 53(94.60%) |  |  |
| Have you ever undergone surgery? |  |  |  | 7.575^a^ | 0.023 |
| Yes | 87(86.10%) | 192(92.80%) | 55(98.20%) |  |  |
| No | 14(13.90%) | 15(7.20%) | 1(1.80%) |  |  |

^a^ Chi-square test.

^b^ Fisher's exact test.

**Fig.S1** Anxiety-depression symptom networks of colorectal cancer patients with and without a stoma.

(Orange line: patients without stoma; Blue line: patients with stoma)

**Fig.S2** Bootstrap edge weight estimation graphs for the patients with anxiety and depression symptoms

This plot corresponds to the Fig 2 (main text).

**Fig.S3** Diagram of the stability of node strength and bridge Expected influence.

This plot corresponds to the Fig 3 (main text).

**Fig.S4** Bootstrap edge weight stability plots for patients with and without a stoma, this plot corresponds to the Fig 4 (main text).

(upper: stoma group; lower: non-stoma group).

**Fig.S5** Stability of centrality plots for patients with and without a stoma, this figure corresponds to Fig S1 (in the supplementary materials section).

(upper: stoma group; lower: non-stoma group).
